# Obstacles in measles elimination: an in-depth description of a measles outbreak in Ghent, Belgium, spring 2011

**DOI:** 10.1186/0778-7367-71-17

**Published:** 2013-07-08

**Authors:** Toon Braeye, Martine Sabbe, Veronik Hutse, Wim Flipse, Lina Godderis, Geert Top

**Affiliations:** 1 Scientific Institute of Public Health, Brussels, Belgium; 2 Flemish Agency for Care and Health, Infectious Disease Control, Ghent, Belgium; 3 Flemish Agency for Care and Health, Brussels, Belgium

**Keywords:** Outbreak, Vaccine-preventable-disease, Measles, Immunization registry, Anti-vaccination

## Abstract

**Background:**

From Mid-February to April 2011 one of the largest measles-outbreak in Flanders, since the start of the 2-dose vaccination scheme in 1995, took place in Ghent, Belgium. The outbreak started in a day care center, infecting children too young to be vaccinated, after which it spread to anthroposophic schools with a low measles, mumps and rubella vaccination coverage. This report describes the outbreak and evaluates the control measures and interventions.

**Methods:**

Data collection was done through the system of mandatory notification of the public health authority. Vaccination coverage in the schools was assessed by a questionnaire and the electronic immunization database ‘Vaccinnet’. A case was defined as anyone with laboratory confirmed measles or with clinical symptoms and an epidemiological link to a laboratory confirmed case. Towards the end of the outbreak we only sought laboratory confirmation for persons with an atypical clinical presentation or without an epidemiological link. In search for an index patient we determined the measles IgG level of infants from the day care center.

**Results:**

A total of 65 cases were reported of which 31 were laboratory confirmed. Twenty-five were confirmed by PCR and/or IgM. In 6 infants, too young to be vaccinated, only elevated measles IgG levels were found. Most cases (72%) were young children (0–9 years old). All but two cases were completely unimmunized. In the day care center all the infants who were too young to be vaccinated (N=14) were included as cases. Thirteen of them were laboratory confirmed. Eight of these infants were hospitalized with symptoms suspicious for measles. Vaccination coverage in the affected anthroposophic schools was low, 45-49% of the pupils were unvaccinated. We organized vaccination campaigns in the schools and vaccinated 79 persons (25% of those unvaccinated or incompletely vaccinated).

**Conclusions:**

Clustering of unvaccinated persons, in a day care center and in anthroposophic schools, allows for measles outbreaks and is an important obstacle for the elimination of measles. Isolation measures, a vacation period and an immunization campaign limited the spread of measles within the schools but could not prevent further spread among unvaccinated family members. It was necessary to raise clinicians' awareness of measles since it had become a rare, less known disease and went undiagnosed.

## Background

Measles, an infectious childhood disease, has re-emerged all over Europe [1]. In the region of Flanders, Belgium, vaccination against measles with the measles-mumps-rubella vaccine (MMR) started in 1985 for children at the age of one year, a second dose was added in 1995 for children at the age of 10 years. Since the start of the two-dose vaccination scheme the disease has only been rarely reported, with the exception of one large measles outbreak in an orthodox Jewish community in Antwerp in 2008, involving 137 cases [2]. Some of their private schools were not attended by school health services. This resulted in a low vaccination coverage.

A European elimination goal was set for 2010 [3], but since 2008 a rise in measles cases has been reported with outbreaks in 36 countries of the WHO European Region [1]. The critical attitude of some communities towards vaccination is one of the reasons for these outbreaks, which often occur in groups of unimmunized people living in a population with a good overall coverage [4-6]. In a new WHO-resolution the commitment to eliminate measles and rubella has been renewed and is now set for 2015 [7].

Vaccination is known to be the main protection against measles. A one-dose effectiveness of at least 95% [8] limits vulnerability almost exclusively to those unvaccinated. Herd immunity is possible at coverage levels higher than or equal to 95%. Flanders has a documented first dose MMR vaccine coverage of 96.6% for toddlers and a second dose coverage of 92.5% for adolescents [9]. These data were collected in 2012 and they present a 1.9% improvement over data collected in 2008 [10]. A better catch-up policy might further improve the vaccination coverage. A seroprevalence study from 2006 demonstrated a seronegativity for measles of 3.9% for all studied ages (1–65 year) [11]. In the age group of 1 to 24 years seronegativity is higher, too high for herd immunity. Despite the high vaccination coverage a susceptibility thus still exists in these age groups [12].

Anthroposophy is a spiritual philosophy based on the teachings of Austrian-born Rudolf Steiner. The two schools most affected in this outbreak offered Steiner education. This is a largely independent, alternative education movement offering a humanistic approach to pedagogy [13].

Since 2011 an important recurrence of measles has been observed in Belgium [14]. Our report describes the largest and best defined cluster of this recurrence, starting in a day care center in Ghent and spreading to anthroposophic schools.

## Methods

We gathered information on patient characteristics (gender, age, family size), symptoms, treatment (if any), the vaccination status and the contact history. A case was defined as anyone with laboratory confirmed measles or anyone with a generalized, maculopapular, erythematous rash and an epidemiological link to a laboratory confirmed measles case. The first cases were reported through the system of mandatory notification. All physician and laboratories have the legal obligation to notify measles cases to the local Infectious Disease Control Unit of the Public Health Surveillance. In the anthroposophic schools data on vaccination, reasons for not being vaccinated and previous measles infection were collected through questionnaires.

Detection of measles virus RNA (nested RT-PCR) and antibodies in oral fluid and serum (IgM detection by Elisa, MicroImmune) were used as laboratory confirmation. Oral fluid and serum samples were sent to the National Reference Center for Measles and Rubella [15]. Elevated measles IgG was accepted as criterion for laboratory confirmation in infants older than 6 months but too young to be vaccinated. Towards the end of the outbreak laboratory testing was only recommended for those with atypical clinical symptoms and those with no known epidemiological link.

In order to document the outbreak in the day care center we explored the medical history and collected oral fluid samples of all infants who, up to two months prior to the outbreak, were part of the youngest group (younger than one year). Seven infants were hospitalized shortly prior to the outbreak investigation, residual blood samples were tested for measles antibodies (IgM). We further enquired into the recent medical history of the parents and the personnel of the day care center, including interns and trainees.

To assess the risk and plan interventions, we calculated the vaccination coverage for all three affected anthroposophic schools. We obtained information on vaccination through a questionnaire distributed to all pupils and through ‘Vaccinnet’. ‘Vaccinnet’ is a web-based vaccine ordering system as well as a computerized immunization registry for Flanders. Up to 92.5% of recent vaccinations are registered in ‘Vaccinnet’ [16,17]. We used school lists to manually extract vaccination data. We calculated the vaccination coverage based on ‘Vaccinnet’ and based on the questionnaire.

## Results

### Course of the outbreak

At the beginning of March 2011, five cases of measles were reported to the Infectious Disease Control Unit of the Public Health Surveillance of East-Flanders. Two cases were brothers, but there was no obvious link between the other cases. All were unvaccinated. Further investigation revealed that they had all been present at the same time on February 22^nd^ in the waiting room of a general practitioner (GP) with an anthroposophic approach. The youngest of the five cases, a 10-month old boy (index patient) consulted the GP with signs of measles. This infant attended a day care center in Ghent. Three of the four other cases, aged 4, 9 and 12 years, went to three different anthroposophic schools.

**Figure 1:**
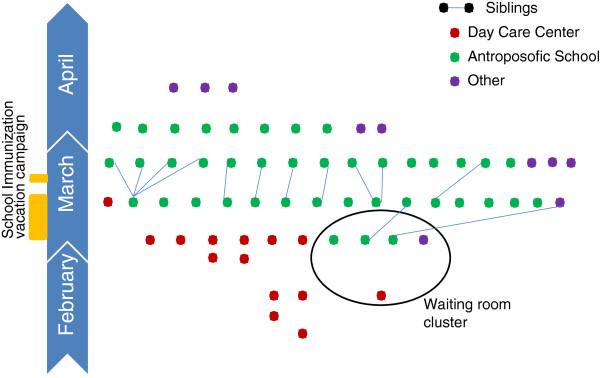
**Overview of the measles outbreak, Ghent, Belgium 2011.** Dots represent cases (N=65). Cases that are siblings are connected by a line. Timeline is only indicative.

New cases (N=16) were reported two weeks later. All these new cases were linked to the “waiting-room”-cases. All but one of these new cases went to school at one of the earlier mentioned anthroposophic schools. The only case not linked through the schools was the sibling of a “waiting-room”-case (Figure 1). In one anthroposophic school the outbreak was limited to one case. In the day care center a new case was reported shortly thereafter.

A new generation of 18 measles cases occurred at the end of March. Eleven of these new cases were family members of known cases. One of the cases without family link was a physician working at an emergency department. She was probably infected during the clinical examination of one of the day care center-cases. In two laboratory-confirmed cases no obvious link with the current cluster could be found. These patients were either part of a different outbreak (one of them, a 24-year old woman, was probably infected during a meeting in Paris) or linked to the outbreak in a way unknown to us.

At the beginning of April, 10 new cases were reported. Eight of these cases were school contacts. Two could not be linked.

**Figure 2:**
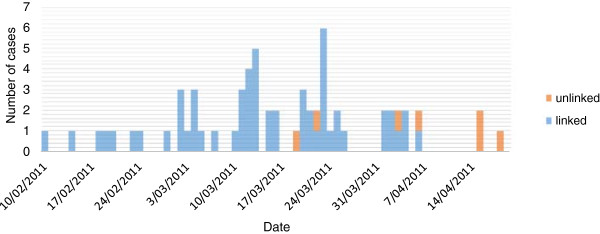
**Epidemic curve, measles outbreak Ghent, Belgium 2011.** Epidemic curve of measles cases, 10/02/2011 to 18/04/2011 Ghent, Belgium (N=65), sorted by starting date of symptoms. Epidemiological linked cases (=blue) had a known contact with another case.

From mid-April on, only a three new cases were reported. These cases were laboratory confirmed but without a clear link to the outbreak. This outbreak consisted of a total of 65 cases (Figure 2).

### Patient characteristics

**Figure 3:**
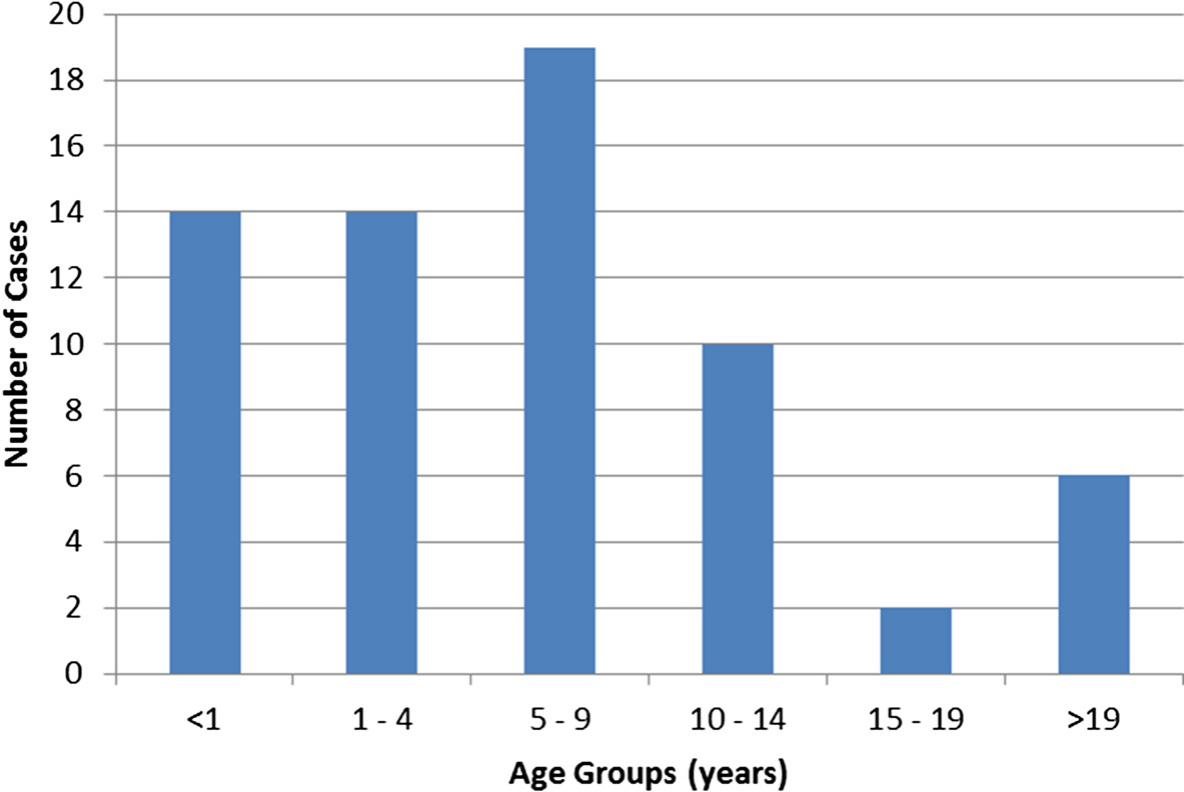
**Age distribution of the cases, measles outbreak Ghent, Belgium 2011.** Number of measles cases by age group (years).

Twenty-eight (=43%) of the 65 cases were females. The median age was 6 years (range 7 months to 27 years). Most cases were between 5 and 10 years old (N=19, 29%) (Figure 3). Infants (< 1 year old) accounted for 22% (N=14) and the age group from 10 to 19 years accounted for 18% (N=12) of all the cases.

The clinical presentation of measles was milder in children than in adults and infants. Children, till the age of 14, were absent from school for an average of 5 to 7 days. Adolescents and adults were absent for 14 up to 21 days from school or work. Eight of the 14 infants were hospitalized during the outbreak. The reasons for hospitalization were most often dehydration and fever. No complications such as pneumonia or encephalitis were reported.

All but two cases (3%) were unvaccinated. These two cases, 13 and 26 years old, had only received one dose of MMR-vaccine. The reported reasons for not being vaccinated were; personal, often anthroposophic, beliefs (72%, N=47), illness at the time of vaccination without catch-up vaccination (3%, N=2) and too young to be vaccinated (22%, N=14).

#### The day care center

The index case was part of a group of 14 infants, between the age of 6 months and 1 year, at a day care center. During the period in which we studied the outbreak one infant was shortly hospitalised for measles. Seven other infants had been hospitalised, from one up to seven days, in the previous month with symptoms like stomatitis, exhaustion, fever, vomiting or rash. During their hospital stay nobody except our index case, was diagnosed with measles. The index case spread the measles virus to three children and one adolescent while sitting in the waiting room of a GP with an anthroposophic practice. The diagnosis of measles was not made during that consultation, but during a later hospital stay. We found laboratory confirmation of measles infection in all tested infants. For only one infant no sample could be obtained, but this infant had also shown symptoms associated with measles. The attack rate in the day care center is estimated at 100%. Further research into the medical history of parents, family and the staff, including trainees and interns, did not point to a possible source for the children. None of them reported having had symptoms compatible with measles.

#### The schools

The schools offered education to children aged 3 to 12 years. One of the schools also offered secondary school (12-18y). There were a total of 804 pupils in the three anthroposophic schools affected in this outbreak. We disturbed a questionnaire to all pupils and received 550 responses (response rate of 68%). The results show that 45% (N=246) of the children were unvaccinated and 14% (N=75) were incompletely vaccinated. These incompletely vaccinated children and adolescents received only the first dose, at the age of one year, and missed or refused the second dose. Based on the vaccination registrations found in ‘Vaccinnet’ 49% were unvaccinated and 17% were incompletely vaccinated. A total of 30 pupils claimed within the questionnaire to have been previously infected with measles virus. In about one third of the questionnaires the reason for not vaccinating their child was described by the parents as “a personal choice”.

### Laboratory testing

The National Reference Center for Measles and Rubella analysed a total of 45 oral fluid samples from suspected measles cases during the outbreak in Ghent. The total number of positive samples at the reference center was 29. Samples from two infants were not tested at the reference center but were found measles IgM positive at another laboratory. The total number of laboratory confirmed cases was 31.

We obtained samples from 13 of the 14 children in the day care center, 3 serum samples and 10 saliva samples. Two serum samples were only tested for measles IgM and both were measles IgM positive. Five, of which one serum sample, were found positive for both measles IgG and IgM. In the remaining six saliva samples no measles IgM could be found but all were positive for measles IgG. PCR genotyping of two oral fluid samples resulted in genotype D4-Hamburg for both.

Aside from the day care center, 34 samples were collected, 18 were positive for measles. Five of these samples had both measles RNA and measles IgM, four were only measles IgM positive and in nine only measles RNA could be detected, no measles antibodies.

The 16 samples in which measles RNA was detected were also genotyped by an in-house developed assay. All were genotyped as D4, subvariant strain MVs/Ghent. BEL/09.11/1/[D4]. This strain was clearly related to MVs/Hamburg.DEU/03.09/ [D4].

### Measures taken

Several control measures were taken to limit the spread of measles. We tried to reduce the number of susceptibles by means of an immunization campaign in the schools. Prior to the campaign all students were given a leaflet with the risks and complications of a measles infection and some information on vaccination. Children with incomplete measles vaccination were offered vaccination at the school, during school hours, by the outbreak team. During the vaccination campaigns on 21, 22 and 23 March 2011, we vaccinated 25% (N=79) of 321 incompletely vaccinated or unvaccinated children.

We raised clinical alertness by informing health care professionals on the outbreak. Several letters and e-mails were sent to emergency departments, GPs and pediatricians. Physicians were made aware of the procedures to obtain free test kits for oral fluid sampling.

We tried to isolate cases from unimmunized persons. Furthermore not only cases were isolated as we also isolated three unimmunized siblings of cases that went to a day care center. This was done to prevent the spread of measles to another day care center.

A meeting was held with one of the general practitioners with an anthroposophic practice. He could agree on the necessity to vaccinate adolescent boys and girls. Mostly because of the severity of measles at an adolescent or older age but also to avoid rubella infection during pregnancy. Other healthcare professionals and some parents did prove more resilient against vaccination. For example: during the immunization campaign we noticed that some leaflets linking MMR-vaccination to autism and allergies were distributed together with our consent forms. The high percentage of parents that stated that not vaccinating their children is a personal choice is another example of resistance against vaccination.

## Discussion

### Measles in infants

The largely undiagnosed spread of measles in a day care center is one of the most important observations in this outbreak. A high attack rate in six to twelve months old infants has previously been described in other outbreaks [18,19]. In this outbreak measles infection in infants was associated with a high morbidity; the hospitalisation of eight infants during or shortly prior to the outbreak investigation was probably due to measles. Elevated IgG levels were found in the oral fluid of 11 infants. In six infants this was not accompanied with elevated IgM levels. These samples were taken during the outbreak investigation, three up to six weeks after the symptoms had subsided. IgG levels indicate either prior infection or persisting maternal antibodies. The latter is highly unlikely for children aged six months and older. At the age of six months 99% of infants of vaccinated mothers are vulnerable. This percentage is slightly lower, 95%, for infants of naturally immune mothers [19,20]. The elevated IgG levels are thus more likely to indicate a previous measles infection. In one of the samples still available from a hospitalisation mid-February we found measles IgM. Both the IgM and the IgG confirm that measles was present in the day care center before the first notification. Measles has been quite rare in Belgium for several years and clinicians failed to diagnose it. Other, more common, diseases, such as exanthema subitum, viral rash (of unknown origin) or stomatitis, were placed higher in the differential diagnosis. This is an important issue since health care associated spread is not uncommon. Different outbreak reports have already described how measles spreads in consultation rooms and emergency departments [21-24].

### Anthroposophic views

The European Council for Steiner Waldorf schools does not disapprove of vaccination, stating that “families provide the proper context for such decisions” [25]. These schools are however internationally known for their low vaccination coverage. In the United Kingdom they are categorized by the Health Protection Agency as “unvaccinated community” [26]. In the anthroposophic schools affected in this outbreak we found a low MMR-vaccination coverage, 45-49% of the pupils were unvaccinated. In 2008 vaccination coverage was compared in schools in Antwerp with different belief systems [27]. Whereas the mainstream schools approached a coverage of 93% for the first dose of MMR, the anthroposophic schools had a one dose coverage of 50%. It is hard to evaluate the success of the immunization campaign. We vaccinated only 25% of the susceptible pupils. Since the response rate of the questionnaire was only 68% and one can expect that parents who accept the offer to vaccinate their children are more likely to fill in the questionnaire, the percentage of susceptible children that were immunized is probably lower. As in previous years, catch-up vaccination will be offered on a regular basis by the school health services.

Despite the low vaccination coverage, the spread of the infection within the schools was limited. We believe that the explanation for this can partly be found in the early isolation of the cases, a school vacation [28] and to a lesser extend in the vaccination campaign. Some natural immunity might have been present within this group, 30 pupils claimed a previous measles infection.

After the spread within the waiting room and the first spread within the schools, the new generation of measles cases consisted mainly of family members. All unvaccinated siblings fell ill 10 to 14 days after measles was introduced in a family, bringing the attack rate for unvaccinated siblings to 100%. We insisted on vaccinating any unvaccinated family members, since isolation is infeasible within families, but limiting the spread of measles within a family presents a huge challenge.

Risks for future outbreaks are still present. A large amount of susceptible children still remain in these anthroposophic schools. As is shown in this outbreak, waiting rooms of GPs with an anthroposophic practice are gathering points for both the ill and the unvaccinated. This can facilitate the spread of infectious, vaccine preventable diseases. A change in the belief of these groups and professionals will be a necessary step to accomplish measles elimination [29]. Attaining an overall vaccine coverage of 95% will not suffice if clusters of unvaccinated persons persevere [30] even if these clusters are small [31]. The policy to exclude unvaccinated students from school during an outbreak has proven to be successful in previous outbreaks [5], but is probably unfeasible in this setting. A large amount of students would not be able to attend classes for a long period and as vaccination is seen as a personal choice, the policy of the schools will not allow for such a dominant approach.

### Microbiology

D4-Hamburg is a new strain of measles virus imported from London, United Kingdom, to Hamburg, Germany, in December 2008 [32]. D4-Hamburg has been present in Europe for more than three years and has led to more than 25,000 cases in 12 countries. Its spread was mainly but not exclusively associated with travelling Roma [33].

We promoted testing on oral fluid over traditional venepuncture. The collection of oral fluid is less invasive, less painful, less expensive (i.e. no trained personnel required) and safer (prevention of needle stick injuries). The serological diagnosis (IgM detection by ELISA, MicroImmune) of measles on oral fluid has a high sensitivity and specificity, respectively 92% and 100% compared to traditional ELISA on serum. Molecular diagnosis (nested RT-PCR) offers a sensitivity and specificity of 100% compared to standard assay on nasopharyngeal secretions [34].

## Conclusions

We report the largest and most remarkable Belgian measles outbreak within the overall recurrence of measles in 2011 and one of the largest outbreaks since the start of the two-dose vaccination scheme. The outbreak consisted of 65 cases of which 63 were unvaccinated. Initially measles went undiagnosed in the day care center. This allowed the disease to spread within the center, finally affecting 14 infants and causing 8 hospitalisations. The large number of susceptible children concentrated in anthroposophic schools was responsible for most of the spread in this outbreak. The clustering of unimmunized persons, due to a critical attitude towards vaccination, will allow for future outbreaks and could be an important obstacle for measles elimination.

## Competing interests

The authors declared that they have no competing interest.

## Authors’ contribution

TB collected data and samples, helped in setting up and performing the vaccination campaigns, constructed and analysed the databank, main author of the article. MS collected measles info on a national level, contributed to the background and discussion sections. VH did all microbiology and wrote the parts on microbiology. WF collected data on a local level, led the vaccination campaign and other control measures. LG collected and summarized local information (including patient contact and sample collection), aided in the vaccination campaign and other control measures. GT worked mostly on Vaccinnet and is a national expert on vaccine preventable diseases. All authors read and approved the final manuscript.
